# Increased COVID-19 Severity among Pregnant Patients Infected with SARS-CoV-2 Delta Variant, France

**DOI:** 10.3201/eid2805.212080

**Published:** 2022-05

**Authors:** Souheil Zayet, Vincent Gendrin, Catherine Gay, Philippe Selles, Timothée Klopfenstein

**Affiliations:** Nord Franche-Comté Hospital, Trevenans, France

**Keywords:** COVID-19, respiratory infections, severe acute respiratory syndrome coronavirus 2, SARS-CoV-2, SARS, coronavirus disease, zoonoses, viruses, coronavirus, pregnancy, France

## Abstract

We conducted a retrospective study of pregnant persons hospitalized for severe acute respiratory syndrome coronavirus 2 infection in France. Delta variant infection had a relative risk of 14.33 for intensive care unit admission and 9.56 for high supplemental oxygen support. The Delta variant might cause more severe illness during pregnancy.

The obstetric practice of Nord-Franche-Comté Hospital, France, has ≈3,600 deliveries per year (*1*). A recent study warned about the possibility of more severe coronavirus disease (COVID-19) among pregnant persons infected with severe acute respiratory syndrome coronavirus 2 (SARS-CoV-2) Delta variant (*2*). In France, the Delta variant became the predominant circulating SARS-CoV-2 variant in late June 2021 (*3*). We explored whether severe COVID-19 cases among pregnant persons increased in our facility when the Delta variant was predominant.

We conducted a retrospective study on all hospitalized pregnant women diagnosed with COVID-19 by reverse transcription PCR of nasopharyngeal swab samples during March 1, 2020–November 15, 2021. We defined severe COVID-19 as a case requiring intensive care unit (ICU) admission and critical COVID-19 as a case in the ICU that required high supplemental oxygen support, either high-flow nasal cannula, noninvasive ventilation, or mechanical ventilation.

We defined the predominant SARS-CoV-2 variants during 3 periods as variants detected in >50% of all sequences analyzed nationwide. National data from epidemiologic surveillance showed that wild-type was the predominant variant until March 1, 2021 (period 1); Alpha (20I/501Y.V1) during March 2–June 28, 2021 (*4*) (period 2); and Delta (21A/478K.V1) during June 29–November 15, 2021 (period 3). Beta (20H/501Y.V2) and Gamma (20J/501Y.V3) variants also were circulating in France but were not predominant. 

To compare the frequency of severe and critical COVID-19 among the 3 periods, we calculated the ratio of women of reproductive age (defined as 15–42 years) hospitalized with COVID-19 during the same period. During March 1, 2020–November 15, 2021, a total of 77 women of reproductive age were hospitalized for COVID-19 in our facility, including 30 pregnant women (Figure). Among the 30 pregnant persons, 7 were transferred to the ICU (1 confirmed Alpha variant, 6 confirmed Delta variant cases), 5 of whom required high supplemental oxygen support (1 Alpha variant, 4 Delta variant cases). None of the 7 severe or critical COVID-19 patients were vaccinated. 

**Figure:**
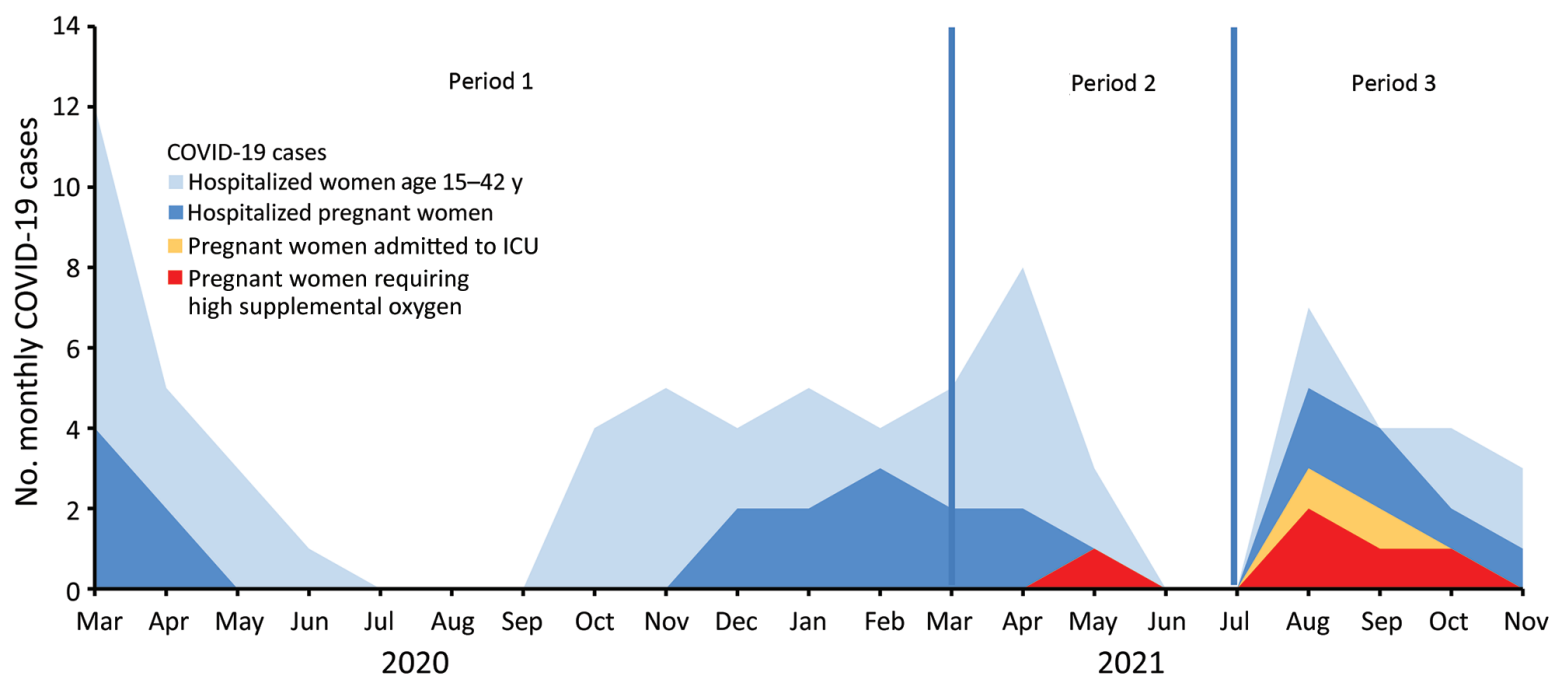
Monthly cases of hospitalized, severe, and critical COVID-19 cases among women of childbearing age (15–42 years) and pregnant women at Nord Franche-Comté Hospital, France, March 1, 2020–November 15, 2021. We assessed COVID-19 disease severity against circulating severe acute respiratory syndrome coronavirus 2 (SARS-CoV-2) variants during 3 periods of interest based on predominance of circulating variants. During period 1, wild-type virus comprised >50% of all sequenced SARS-CoV-2 variants in France; during period 2, >50% were Alpha variant; and during period 3, >50% were Delta variant. COVID-19, coronavirus disease; ICU, intensive care unit.

For each period, we calculated the ratio between severe and critical COVID-19 among pregnant women and all women of reproductive age hospitalized for COVID-19. For period 1, the ratio was <2.33% (0 severe cases; thus, <1 among 43 cases); for period 2, 6.25% (1 severe case/16 cases); and for period 3, 33.33% (6 severe cases/18 cases). The ratio between pregnant women with critical COVID-19 and all women of reproductive age hospitalized for COVID-19 was <2.33% (0 critical cases; thus, <1 among 43 cases) for period 1; 6.25% (1 critical case/16 cases) for period 2; and 22.22% (4 critical cases/18 cases) for period 3.

Based on these ratios, compared with period 1, the relative risk for ICU admission was 2.69 (95% CI 0.18–40.46) for period 2 and 14.33 (95% CI 1.86–110.70) for period 3. The relative risk for high supplemental oxygen support was 2.69 (95% CI 0.18–40.46) for period 2 and 9.56 (95% CI 1.15–79.70) for period 3.

The risk ratios for severe and critical COVID-19 during the 3 periods rebut the hypothesis that the increasing number of SARS-CoV-2 infections in younger persons, combined with low acceptance for COVID-19 vaccination during pregnancy, sufficiently explain the increased risk for severe disease noticed with the Delta variant (*5*). SARS-CoV-2 lineage B.1.617 (Delta) probably is associated with increased COVID-19 severity among pregnant persons compared with previous variants (*2*,*6*). This consistent difference suggests a change in pathogenicity in pregnant persons and requires further investigation. A large retrospective cohort study comparing similar groups of pregnant women with COVID-19 during the pre-Delta period (n = 224) and the Delta period (n = 69) suggested an increase in critical illness and adverse perinatal outcomes associated with the Delta variant during pregnancy (*7*). Another study showed that pregnant patients infected with the Delta variant were more symptomatic and were diagnosed earlier than patients diagnosed before Delta was prevalent (*8*). Our results support the possibility of increased COVID-19 severity with Delta compared with previous SARS-CoV-2 variants. 

Our study’s first limitation is that standard care and hospitalization criteria changed between the 3 periods, which could have affected our results. We suspect thresholds for ICU admission were lower for pregnant persons during periods 2 and 3 than during period 1 because of a partial ICU bed saturation during the first COVID-19 wave (*9*). COVID-19 treatment progressively improved and standard care was more optimal during periods 2 and 3 than period 1 (Appendix); thus, we should have expected fewer severe and critical COVID-19 patients in periods 2 and 3, but we observed the opposite. The main limitation of our study is the small sample size in a monocentric study, which prevents us from issuing any conclusions. 

Despite the small number of cases, our findings on COVID-19 severity among pregnant persons infected with the Delta variant are consistent with those of other studies (*2*,*6**–**8*). A larger national cohort study, such as the one conducted by the UK Obstetric Surveillance System (N. Vousden et al., unpub. data, https://www.medrxiv.org/content/10.1101/2021.07.22.21261000v1), could confirm our findings. Nonetheless, our results show that SARS-CoV-2 prevention measures, especially COVID-19 vaccination, are needed during pregnancy.

Appendix Additional treatment information for pregnant persons infected with SARS-CoV-2 at risk for severe COVID-19.
