# Supplementary material for: Increased COVID-19 Severity among Pregnant Patients Infected with SARS-CoV-2 Delta Variant, France
Source: Emerg Infect Dis. 2022 May;28(5):1048–50. doi: 10.3201/eid2805.212080 (PMC9045421; doi:10.3201/eid2805.212080)
Supplement: Appendix — Additional treatment information for pregnant persons infected with SARS-CoV-2 at risk for severe COVID-19. [file 21-2080-Techapp-s1.pdf]

Article DOI: https://doi.org/10.3201/eid2805.212080

Increased COVID-19 Severity among Pregnant Patients Infected with SARS-CoV-2 Delta Variant, France

Appendix

Additional Conclusions

Pregnant persons with coronavirus disease (COVID-19) should receive low molecular weight heparin (LMWH) and proven therapies, such as corticosteroids and tocilizumab, on the basis of risk/benefit according to disease severity (*1*). Corticosteroids and tocilizumab were retained as proven therapies for COVID-19 in RECOVERY study results from June 2020 (*2*) and February 2021 (*3*). For these reasons, we think that during the first part of period 1 (up to March 1, 2021), standard care was less optimal than the other periods in our study (March 2–November 15, 2021).

References

1. Nana M, Nelson-Piercy C. COVID-19 in pregnancy. Clin Med (Lond). 2021;21;e446–50. PubMed https://doi.org/10.7861/clinmed.2021-0503

2. Sterne JAC, Diaz J, Villar J, Murthy S, Slutsky AS, Perner A, et al.; WHO COVID-19 Clinical Management and Characterization Working Group. Corticosteroid therapy for critically ill patients with COVID-19: A structured summary of a study protocol for a prospective meta-analysis of randomized trials. Trials. 2020;21;734. PubMed https://doi.org/10.1186/s13063-020-04641-3

3. RECOVERY Collaborative Group. Tocilizumab in patients admitted to hospital with COVID-19 (RECOVERY): a randomised, controlled, open-label, platform trial. Lancet. 2021;397;1637–45. PubMed https://doi.org/10.1016/S0140-6736(21)00676-0
